# Edinburgh Medico-Chirurgical Society

**Published:** 1888-02

**Authors:** 


### EDINBURGH MEDICO-CHIRURGICAL SOCIETY.

NOVEMBER 16, 1887.

The President, Dr. John Smith, in the Chair.

### THE ÆTIOLOGY OF TUMORS.

Dr. SIMS WOODHEAD, in introducing a discussion on this subject, referred to certain observations which he had published some three years before, and remarked that the intervening time had only strengthened his previous conclusions. The doctrines first promulgated by Cohnheim were not sufficient to explain all the facts with which they had to deal. A careful study was rehearsed of the growth and development of the ovum, and the changes undergone by the different layers of the blastoderm. Some form of irritation or stimulation was necessary for the segmentation process. When this irritation became proportionately greater than the nutrition afforded, reproduction occurred without specialization. The tissues derived from each of the three layers of the blastoderm differed in respect of rate of development, and their several anabolic and catabolic phases were not contemporaneous. Hence the chances of disturbance were greater at different times in each of the groups. This suggested an explanation of the fact of the greater frequency in young subjects of tumors of the connective—tissue type, in adult life of mixed non-malignant tumors, and in old age of tumors of the epithelial type. While imperfect nutrition undoubtedly led to cell proliferation, the presence of an irritant in some form was to be postulated. They had also to recognize the existence of a hereditary tendency to tumor production, the special type depending on the age of the individual's parents, and the state of equilibrium of their tissues at the date of the individual's conception. Such a tendency, accentuated by passage through a succession of generations, might ultimately result in the establishment of a diathesis.

Professor CHIENE cited a large number of interesting cases in illustration of the thesis. He maintained that malignant tumors had their origin in some local irritation or injury. They must give the word irritant a wide significance.

Dr. JAMES was inclined to agree with the view advanced by Dr. Woodhead, and congratulated him on the biological soundness of his carefully elaborated generalizations.

Dr. DUNCAN said in such a discussion they must separate clearly between simple tumors proper and malignant growths. The former had their explanation in teratology, while malignant tumors were distinctively intrusive. They were probably zymotic in origin, and had their analogies in inflammation, tubercle, and syphilis. Probably it would be found that they were due to the action of micro-organisms, as had been fairly well demonstrated in the case of the three processes to which he thought them analogous.

Dr. BELL spoke of the clinical significance of injury, and referred to the popular idea that, for example, in the case of the mamma, tumor-formation was traceable to a blow or other injury.

